# Self-Reported Health Status in Primary Health Care: The Influence of Immigration and Other Associated Factors

**DOI:** 10.1371/journal.pone.0038462

**Published:** 2012-06-04

**Authors:** Miguel Á. Salinero-Fort, Rodrigo Jiménez-García, Laura del Otero-Sanz, Carmen de Burgos-Lunar, Rosa M. Chico-Moraleja, Carmen Martín-Madrazo, Paloma Gómez-Campelo, on behalf of The Health & Immigration Group

**Affiliations:** 1 Fundación de Investigación Biomédica, Hospital Carlos III, Servicio Madrileño de Salud, Madrid, Spain; 2 Departamento de Medicina Preventiva, Universidad Rey Juan Carlos, Madrid, Spain; 3 Servicio de Medicina Preventiva, Hospital Universitario Insular-Materno Infantil, Las Palmas de Gran Canaria, Spain; 4 Unidad de Epidemiología Clínica, Hospital Carlos III, Servicio Madrileño de Salud, Madrid, Spain; 5 Unidad de Medicina Interna, Hospital Central de la Defensa Gómez Ulla, Servicio Madrileño de Salud, Madrid, Spain; 6 Centro de Salud Monóvar, Servicio Madrileño de Salud, Madrid, Spain; Public Health Agency of Barcelona, Spain

## Abstract

**Objective:**

The aims of this study are to compare self-reported health status between Spanish-born and Latin American-born Spanish residents, adjusted by length of residence in the host country; and additionally, to analyse sociodemographic and psychosocial variables associated with a better health status.

**Design:**

This is a cross-sectional population based study of Latin American-born (n = 691) and Spanish-born (n = 903) in 15 urban primary health care centres in Madrid (Spain), carried out between 2007 and 2009. The participants provided information, through an interview, about self-reported health status, socioeconomic characteristics, psychosocial factors and migration conditions. Descriptive and multiple logistic regression analyses were conducted.

**Results:**

The Spanish-born participants reported a better health status than the Latin America-born participants (79.8% versus 69.3%, p<0.001). Different patterns of self-reported health status were observed depending on the length of residence in the host country. The proportion of immigrants with a better health status is greater in those who have been in Spain for less than five years compared to those who have stayed longer. Better health status is significantly associated with being men, under 34 years old, being Spanish-born, having a monthly incomes of over 1000 euros, and having considerable social support and low stress.

**Conclusions:**

Better self-reported health status is associated with being Spanish-born, men, under 34 years old, having an uppermiddle-socioeconomic status, adequate social support, and low stress. Additionally, length of residence in the host country is seen as a related factor in the self-reported health status of immigrants.

## Introduction

In Spain, immigration has doubled since the second half of the nineties, resulting in a new socio-political reality with consequent associated social and health challenges [1], [2]. Currently, Spain’s population has grown to more than 46 million people, and almost 12% (17% in Madrid) of this growth is accounted for by immigration, not including illegal immigration by foreign-born residents [3]. In 2010, between 60%–80% of foreigners in the north-eastern part of Madrid, came from Latin America (Ecuador, Colombia, Peru, Bolivia, Dominican Republic, and Paraguay); this data is very similar to that reported in other large cities in Spain [3], [4].

Migration involves major changes in a person’s environment, with the incorporation of a new physical, institutional and sociocultural context, so, it may be a stressful experience in itself [5]. Also, foreign-born people encounter many problems in their host country -job insecurity, legal instability, difficulty accessing housing, social isolation and ethnic prejudice [6]; along with major environmental changes to their environment, which may pose a risk to health and affect adaptation and integration in the new society [7].

Self-reported health status has been proposed as a robust and well validated predictor of mortality [8] and evidence also suggests differences between the health status of immigrant and native communities [9]. Research has shown the relationship between immigration and self-reported health status, and how to identify the scope of these relationships and potential mediating variables involved [10], [11].

Latin American immigrants tend to have good health upon their arrival to the host country [12]; this is surprising given the association of poor health with low socioeconomic status [13]. However, this paradox disappears over time, as the health advantage of Latin American immigrants appears to decline with acculturation [12]. The explanation for this phenomenon is unclear and many factors have been associated with impaired perception of health, such as: stress, traumatic exposure in their homeland, separation from family, poverty, low educational levels, lower status jobs, and discrimination all increase the risk for a more negative self-reported health status [14].

The aims of this study are to compare self-reported health status between Spanish-born and Latin American-born adults, who have visited a primary health care setting. Furthermore, we will assess the differences between the Latin American-born communities according to the length of residence in Spain. Additionally, sociodemographic variables and psychosocial factors (social support and stress) associated with a better health status are analysed.

## Methods

### Sample

Twenty primary health care centres in the north-eastern area of Madrid (Spain) were invited to participate. None of these centres had specific programmes for migrants. Before the study began five of these centres preferred not to participate. From each participating primary health care centre we recruited a sample of patients who had visited the primary health care centre for a medical or nursing appointment between the period of January 2007 to December 2009. The number of patients recruited for each subgroup in each centre ranged between 75 and 125. Using a simple random selection method 3000 outpatients were selected.

The interviews were performed by two psychologists who both received homogeneous training in interview methods and the evaluation procedure used in the study in order to minimise interview bias between them.

Interviewers explained the aim of the study to potential participants and invited them to participate. Eligible patients were invited to learn more about the study in a private room, and all interviews were performed after the patient’s clinical visit. Inclusion criteria were: outpatients between 18–55 years of age, who visited for a medical or nursing consultation, and Latin America-born Spanish residents or Spanish-born. Exclusion criteria were assessed by the interviewers according to their clinical judgment. These were: psychotic or bipolar disorder, severe chronic diseases or significant physical/cognitive disabilities that might invalidate informed consent or interview.

The study was approved by the Institutional Review Board of the Ramón y Cajal Hospital (Madrid), and an informed consent form was signed by all participants.

### Variables

The outcome variable was self-reported health status, measured by a single-item self-reported indicator: “Would you say your health in general is…?”. Five response categories were combined into two: poor and fair, good, very good and excellent, as suggested by other authors [9], [15].

Social support was assessed using the Spanish version of the Medical Outcomes Study-Social Support Survey (MOS-SSS) [16], [17]. This is a brief, self-administered and multidimensional survey comprised of twenty items. The first item on the scale assesses network size with the question: “About how many close friends and close relatives do you have (people you feel at ease with and can talk to about what is on your mind)?”. Items two to twenty were scored according to the Likert scale ranking ranging from 1 (never) to 5 (always). The scale contains a global dimension and four sub-dimensions of social support: a) emotional/informational support, the expression of positive affect, empathetic understanding and the encouragement of expression of feelings/the offering of advice, information, guidance or feedback (items: 3, 4, 8, 9, 13, 16, 17 and 19); b) positive social interaction support, the availability of other persons for diversion (items: 7, 11, 14 and 18); c) affective support, love expressions (items: 6, 10 and 20); and d) instrumental support, availability of material aid or behavioural assistance (items: 2, 5, 12 and 15). The scale was transformed into a 0–100 scale, and higher scores indicate high social support [16]. The score was dichotomized, with low-normal (below 75^th^ percentile) and high social support (above 75^th^ percentile) [18]. Cronbach’s Alpha for the total scale was 0.97.

Stressful life events were measured using the Spanish version of the Social Readjustment Rating Scale (SRRS) [19], [20]. This scale includes a list of 43 items that look at high-stress vital events in the past year. Stress is defined as a global score of over 150 [21]. Cronbach’s Alpha was 0.89.

The following sociodemographic variables were measured using a self reporting questionnaire: gender, age (years), country of origin (Spanish-born, Latin American-born), marital status (single, married, divorced, widowed), education level (no studies, primary school, high school, diploma degree, Bachelor’s degree), occupational status (management position, administrative officer/self employed/supervisor, manual skilled worker/unskilled worker, unemployed), economic status (monthly income: less than 500 euros, 500–1000 euros, more than 1000 euros), and length of residence in Spain (always for Spanish-born participants, and in Latin American-born participants: less than five years, and five or more years of residence). Additionally, we collected some specific data about their situation before migration from Latin America-born participants: occupational status, exposure to different types of violence, reasons and conditions for migration and length of residence in Spain (years).

The sample size was calculated taking into account the expected prevalence of the better self-reported health status (good, very good and excellent), obtained after a pilot study in both populations. The results of this pilot study gave the following results: 70% of Latin American-born participants rated their health as good, very good or excellent as did 78% of Spanish-born participants. The following assumptions were also taken into account: 2.75% (Spanish-born) and 3.5% (Latin American-born) precision, 95% confidence interval (95% CI), 20% beta risk and 5% loss. Calculated size was obtained by the IMIM (Municipal Institute for Medical Research) computer program GRANMO 5.2; this was 909 subjects in Spanish-born and 689 in Latin American-born.

**Figure 1:**
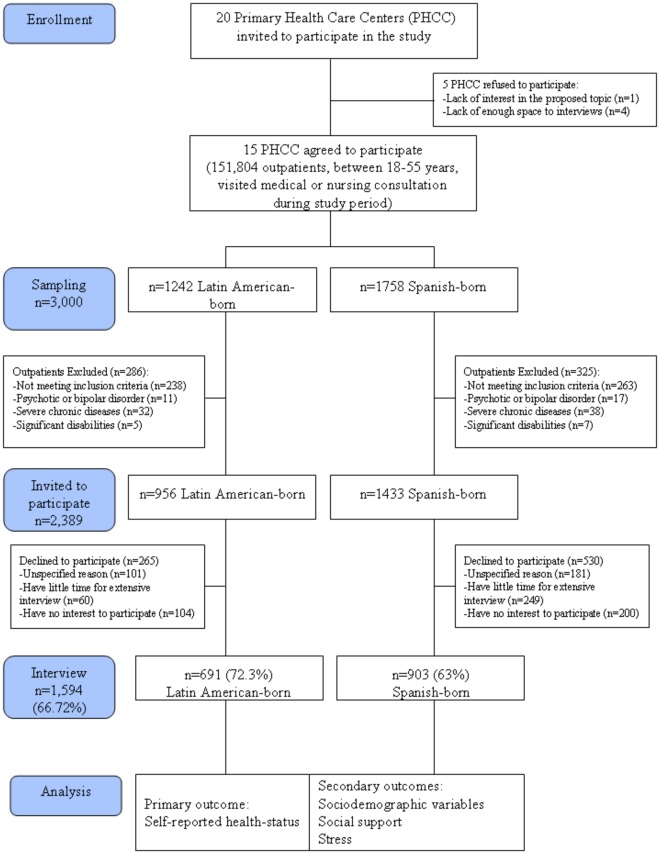
Flow diagram of participants.

### Statistical Analysis

Descriptive statistical analysis was conducted for all the study variables, including the mean and standard deviation (SD) for the quantitative variables, and frequencies for the qualitative variables. The frequency distributions of the qualitative variables were calculated, analysing whether significant differences exist between the two study populations. For the bivariate comparison of proportions, Pearson’s chi-square method or the Fisher exact test method was applied. Student’s t-test was applied for the bivariate comparison of means.

Multivariate analysis was performed to examine the relation of migration status and self-reported health status, using a logistic regression model adjusted by socio-demographic (age, gender, marital status, occupational status, and monthly income) and psychosocial covariates (social support and stress). Variables were introduced in the model step by step based on statistical significance in the bivariate analysis and the adjustment variables considered to be clinically relevant. The model generated (Model 1) contained all variables at a level of p≤0.20 in bivariate analyses, adjusted for confounding factors. The magnitude association is expressed with the Odds Ratio, interpreted as the prevalence ratio (PR). The interactions between country of origin, gender and socioeconomic factors were also checked. Finally, we performed a multivariate analysis (Model 2) that included the variable length of residence in Spain codified into three categories. In all cases, the accepted level of significance was 0.05 or less, with 95% CI.

The statistical analysis was performed using the Statistical Package for Social Sciences (SPSS for windows, version 19.0; Chicago, Illinois, USA).

**Table 1:** Distribution of sociodemographic characteristics of the study population, stratified by country of origin.

	Total(N = 1594)	Latin American-born(n = 691)	Spanish-born(n = 903)	p-value
**Age** (years)**,** mean (SD^a^)	35.9 (10.7)	34.4 (9.7)	37.1 (11.3)	<0.001
**Sex,** % (n)				
Women	66.7 (1063)	59.8 (413)	72.0 (650)	<0.001
Men	33.3 (531)	40.2 (278)	28.0 (253)	
**Marital status,** % (n)				
Single	40.2 (641)	38.6 (266)	41.5 (375)	0.406
Married	50.8 (810)	51.9 (358)	50.1 (452)	
Divorced	702 (115)	8.1 (56)	6.5 (59)	
Widowed	1.7 (27)	1.4 (10)	1.9 (17)	
**Educational level, %** (n)				
No studies	0.6 (10)	0.9 (6)	0.4 (4)	<0.001
Primary school	29.4 (468)	31.9 (220)	10.6 (96)	
High school	32.6 (519)	40.1 (277)	43.6 (394)	
Diploma degree	20.8 (332)	14.3 (99)	25.8 (233)	
Bachelor degree	16.6 (264)	12.8 (88)	19.5 (176)	
**Occupational status, %** (n)				
Manager	8.5 (124)	1.4 (8)	12.8 (116)	<0.001
Administrative/Self employer	25.1 (366)	12.2 (68)	33.0 (298)	
Manual worker	42 (613)	66.2 (368)	27.1 (245)	
Unemployed	24.4 (356)	20.1 (112)	27.0 (244)	
**Monthly incomes, %** (n)				
<500 €	6.6 (106)	11.4 (79)	3.0 (27)	<0.001
500–1000 €	34 (542)	53 (366)	19.5 (176)	
>1000 €	59.4 (946)	35.6 (246)	77.5 (700)	

a: Standard deviation.

**Figure 2:**
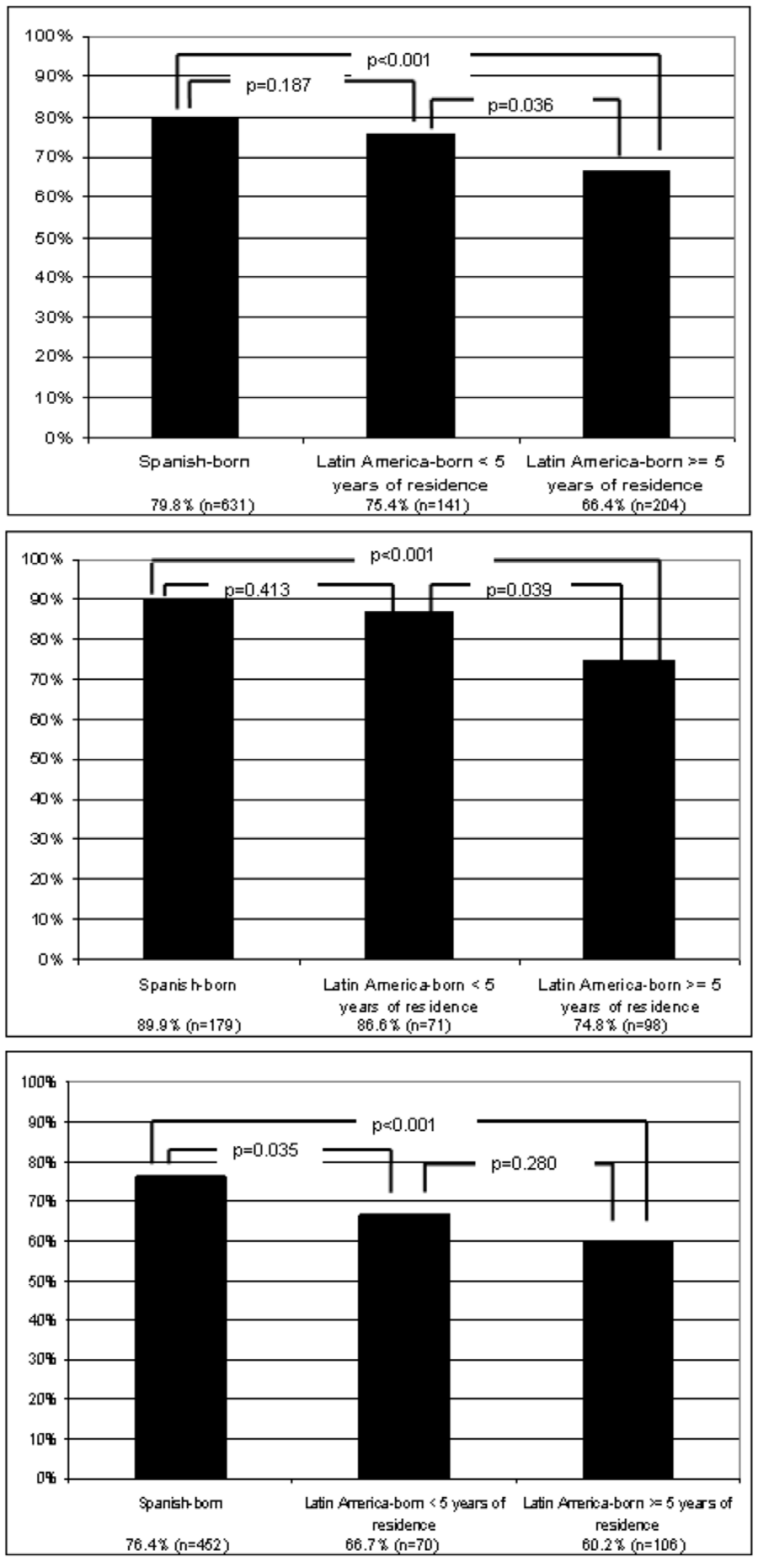
Distribution of good, very good or excellent self-reported health status, of Spanish-born and Latin America-born (stratified by length of residence in the host country), in the total sample (A, top), in men (B, middle), and in women (C, bottom).

## Results

Selection criteria for inclusion in the study were met by 2,389 patients (956 Latin American-born and 1,433 Spanish-born), who were invited to participate (Figure 1). A total of 1,594 subjects voluntarily participated, 691 Latin American-born and 903 Spanish-born, giving an overall response rate of 66.7% (72.3% and 63%, respectively). The origin of the foreign population was 43.3% Ecuadorean, 15.3% Peruvian, 14.5% Colombian, 8.7% Dominican, 6.4% Bolivian and the rest (11.8%) came from different Latin American countries.

**Table 2:** Distribution of psychological variables of the study population, stratified by country of origin.

	Total(N = 1594)	Latin American-born(n = 691)	Spanish-born(n = 903)	p-value
**Social Support** a **,** mean (SD^b^)				
Network size	7.70 (6.90)	6.12 (6.62)	8.91 (6.87)	0.001
Global Support	75.5 (23.7)	68.2 (26.6)	81.5 (19.5)	<0.001
Emotional/Informational Support	74.9 (25.3)	67.1 (28.3)	80.8 (21)	<0.001
Positive Social Interaction Support	76.6 (25.3)	69.9 (28.2)	81.7 (21.6)	<0.001
Affective Support	82.5 (24.6)	77.6 (27.7)	86.7 (21)	<0.001
Instrumental Support	71.6 (29.5)	62.3 (32.3)	78.8 (24.9)	<0.001
**Stress** c, % (n)				
Yes	49.5 (722)	55.9 (310)	45.6 (412)	<0.001

a Score of the Medical Outcomes Study-Social Support Survey (MOS-SSS);

b Standard deviation;

c Score of the Social Readjustment Rating Scale (SRRS).

Statistically significant differences between the Latin American-born and the Spanish-born appear in terms of age, gender, educational level, occupational status and monthly income. These differences show that immigrants are younger, have a lower educational level, work in less skilled jobs and have a lower monthly-income (Table 1).

**Table 3:** Distribution of good/very good/excellent self-reported health status (%) with the crude prevalence ratio, stratified by sociodemographic characteristics, social support and stress.

	%	p-value	CPR^a^ (95% CI^b^)	p-value
**Gender**				
Women (n = 1063)	71.7	<0.001	1	
Men (n = 531)	84		2.07(1.53–2.78)	<0.001
**Age**				
≥34 years (n = 823)	67.8	<0.001	1	
<34 years (n = 771)	84.1		2.51 (1.92–3.28)	<0.001
**Country**				
Latin American born (n = 691)	69.3	<0.001	1	
Spanish born (n = 903)	79.8		1.75 (1.35–2.25)	<0.001
**Marital status**				
Widowed (n = 22)	55	0.02	1	
Divorced (n = 115)	62.4		1.36 (0.50–3.62)	0.545
Married (n = 810)	75.9		2.57 (1.04–6.32)	0.039
Single (n = 641)	78.1		2.92 (1.18–7.21)	0.020
**Educational level**				
No studies (n = 10)	62.5	<0.001	1	
Primary School (n = 468)	66		1.17 (0.27–4.96)	0.834
High School (n = 519)	76.1		1.91 (0.44–8.12)	0.382
Diploma degree (n = 332)	81.2		2.60 (0.60–11.21)	0.201
Bachelor degree (n = 264)	84.1		3.19 (0.72–13.91)	0.124
**Occupational status**				
Unemployed (n = 356)	71.1	<0.001	1	
No qualified worker (n = 613)	70.7		0.98 (0.71–1.34)	0.901
Self employer/Supervisor/Manual qualified worker (n = 366)	83.4		2.05 (1.38–3.02)	<0.001
Manager (n = 124)	85.8		2.47 (1.35–4.48)	0.003
**Monthly income**				
<500 euros (n = 106)	52.3	<0.001	1	
500–1000 euros (n = 542)	66.4		1.80 (1.06–3.06)	0.029
>1000 euros (n = 946)	81.8		4.10 (2.44–6.87)	<0.001
**Social support**				
Low-normal (n = 1180)	72	<0.001	1	
High (n = 414)	84.7		2.42 (1.56–2.94)	<0.001
**Stress**				
Yes (n = 722)	69.5	<0.001	1	<0.001
No (n = 736)	81.2		1.89 (1.44–2.48)	

CPR^a^: Crude prevalence ratio; 95% CI.

b : 95% Confidence Interval.

Data shows that the vast majority of immigrants (86.4%) had a legal status (with residency and/or work permission or nationality), of which a 23.1% reported having Spanish nationality. Economic aspects, family reunification and the search for freedom or new challenges are the most frequently cited reasons for migration (65.1%, 12.4% and 12.2%, respectively). The mean length of residence in Spain was 6.5 years (SD = 4.6 years), and 41.9% of Latin American-born participants had stayed for more than five years. With regard to occupational status before migration, 39.6% were administrative/self employed, 31.9% manual workers, 22.9% unemployed and 6% managers. Being a victim of political violence was reported by 5.8% of these participants, and of family violence in their country by 7.8%. With respect to conditions for migration, the higher values were for migrating alone (69%), with a partner but without children (7.5%) and the rest with other family members.

**Table 4:** Multivariate analysis of good/very good/excellent self-reported health status.

	Model 1		Model 2	
	R^2^ Nagelkerke: 19.4%		R^2^ Nagelkerke: 20%	
	Hosmer-Lemeshov: p = 0.912		Hosmer-Lemeshov: p = 0.858	
	PR ^a^ (95% CI b)	p-value	PR ^a^ (95% CI b)	p-value
**Country of origin**				
Latin American born	1			
Spanish born	1.49 (1.06–2.10)	0.021		
**Length of residence in Spain**				
Always (Spanish-born)			1	
Lower than 5 years (Latin America-born)			1.09 (0.63–1.89)	0.760
5 or more years (Latin America-born)			0.578 (0.40–0.83)	0.003
**Gender**				
Women	1		1	
Men	2.24 (1.48–3.37)	<0.001	2.23 (1.48–3.36)	<0.001
**Age**				
≥34 years	1		1	
<34 years	2.46 (1.75–3.43)	<0.001	2.34 (1.67–2.28)	<0.001
**Marital status**				
Single	1		1	
Married	1.11 (0.78–1.57)	0.547	1.11 (0.78–1.57)	0.566
Divorced	0.93 (0.52–1.65)	0.815	0.93 (0.52–1.64)	0.787
Widowed	0.93 (0.33–2.55)	0.880	0.90 (0.33–2.49)	0.840
**Educational level**				
No studies	1		1	
Primary school	0.65 (0.14–3.07)	0.627	0.71 (0.12–4.17)	0.704
High School	0.86 (0.14–4.90)	0.861	0.96 (0.16–5.64)	0.959
Diploma degree	0.96 (0.16–5.58)	0.960	1.05 (0.18–6.33)	0.955
Bachelor degree	1.24 (0.21–7.35)	0.812	1.31 (0.22–7.98)	0.771
**Occupational Status**				
Unemployed	1		1	
No qualified worker	1.03 (0.73–1.46)	0.710	1.02 (0.71–1.46)	0.915
Self employer/Supervisor/Manual qualified worker	1.46 (0.94–2.24)	0.088	1.50 (0.97–2.13)	0.067
Manager	1.28 (0.64–2.53)	0.487	1.32 (0.66–2.63)	0.428
**Monthly income**				
<500 euros	1		1	
500–1000 euros	1.29 (0.67–2.45)	0.442	1.32 (0.69–2.52)	0.404
>1000 euros	1.96 (1.03–3.80)	0.046	2.06 (1.06–4.00)	0.034
**Social support**				
Low-normal	1		1	
High	1.80 (1.26–2.57)	0.001	1.82 (1.27–2.61)	0.001
**Stress**				
Yes	1		1	
No	2.05 (1.51–2.76)	<0.001	2.06 (1.53–2.77)	<0.001

PR **^a^**: Prevalence ratio; 95% CI.

b : 95% Confidence Interval.

Data on Latin American-born participants, stratified by gender, showed that there were no statistically significant differences in sociodemographic or psychosocial variables, except that, compared to men, women had poorer social support (14.8% versus 28.8%, p<0.001), were more frequently single (35.6% versus 43%, p = 0.005), not working (21.8% versus 15.4%, p = 0.046) and with incomes of under 500 euros (14% versus 7.6%, p = 0.006).

With respect to self-reported health status, Spanish-born participants had better health status (good, very good or excellent) than Latin American-born participants (79.8% versus 69.3%, p<0.001). The sample of Latin American-born participants stratified by length of residence in Spain as in the overall sample, and this was compared to the sub-sample of men and women. This data showed a descending gradient of health status among Spanish-born participants, followed by Latin American-born participants with less than five years residence and Latin American-born participants with a longer duration of residence in Spain (Figures 2A, 2B and 2C).

In the overall sample, as in both the samples for men and women, the difference was significant between Spanish-born and Latin American-born participants who had resided in Spain for five years or more. We did not find significant differences between Spanish-born and Latin-American born who have less than five years of residence in Spain, except in the subsample of women. A significant difference was observed between women Spanish-born and Latin American-born participants with residence of less than five years, although this was not observed for equivalent men participants.

Differences in perception of social support were found between the two groups analysed. Spanish-born participants showed better global, emotional, instrumental, social interaction and affective support than Latin American-born participants (Table 2). As to social network size, the group of Latin American-born participants reported having a smaller network size than those Spanish-born (6.1 and 8.9, respectively), showing a statistically significant difference (p = 0.001). Regarding the percentage of subjects with stress in the sample, Latin American-born participants reported significantly (can only say significantly if statistically significant, otherwise can say considerable more) more stress (55.9%) than those Spanish-born (45.6%).

Overall 75.6% (n = 1594) or participants reported good, very good or excellent self-reported health status. Respondents reporting this were more likely to be men, under 34 years old, Spanish-born, single or married, having a Bachelor’s degree, working as managers or manual skilled workers, having a monthly income of over 500 euros, with high social support and low stress (Table 3).

Multivariable analysis (Model 1) confirms significant differences in: positive self-reported health status in men (PR = 2.24, 95% CI = 1.48–3.37), under 34 years of age (PR = 2.46, 95% CI = 1.75–3.43), Spanish-born (PR = 1.49, 95% CI = 1.06–2.10), with monthly incomes of over 1000 euros (PR = 1.96, 95% CI = 1.03–3.80), with high social support (PR = 1.80, 95% CI = 1.26–2.57), and no stress (PR = 2.05, 95% CI = 1.51–2.76) (Table 4).

Finally, the multivariate analysis (Model 2) that included the variable length of residence in Spain (three categories) confirms the results of previous multivariable analyses with similar PR values for the variables, showing that self-reported health status in Spanish-born participants is similar to Latin American-born participants who have resided in Spain for less than 5 years (PR = 1.09, 95% CI = 0.63–1.89). Furthermore, this data shows that being Latin American-born and living in Spain for 5 or more than 5 years is associated with a poorer health status (PR = 0.58, 95% CI = 0.40–0.83).

## Discussion

This research provides evidence that Latin American-born residents in Spain have a poorer self-reported health status compared with Spanish-born residents. These results are consistent with earlier studies that showed differences in self-reported health status between immigrants of different countries and natives, showing poorer health status among the foreign-born [22]–[24].

In contrast, some researchers suggest no significant health differences exist when comparing natives versus those that are foreign-born, confirming the effect of the “healthy migrant”. However, differences appear when data for the foreign born sample is adjusted for length of residence in the host country. Once adjusted this data shows that the health status of immigrants on arrival is good, and similar to that of the native-born, but it declines over time [25]–[27]. Results of our study provide support for the healthy immigrant paradox. However, when stratified by gender, this phenomenon does not occur in women. Instead, we found significant differences between women Spanish-born participants and women Latin American-born participants who had been living in Spain for less than five years. A significant difference is not found when the two groups of women Latin American-born participants are compared. The disparity in results may be explained by the fact that Latin American-born women had a poorer socioeconomic status (were more frequently not working and had incomes of less than 500 euros), and poorer social support than men, which replicated data found by Aerny et al. [25].

Therefore, the results confirm that, self-reported health status in the Spanish-born participants is similar to that of Latin American-born participants who have been residing in Spain for less than five years. Moreover, Latin American-born participants who have been living in Spain for more than five years have a poorer self-reported health status than Spanish-born participants, after adjusting by sociodemographic and psychosocial variables (Model 2).

The rapid decline of health status occurring over a short period of time (five years), suggests that changes in self-reported health status can be explained by the decline of optimism and the socioeconomic and cultural reality of immigrant life in the host country. This, together with the lack of a social network and family support, can have an impact on a person’s self-reported health status [23]. Therefore, it is necessary to consider the simultaneous influence of sociodemographic and psychosocial variables when considering better health reported health statuses. Data from the multivariate analysis that did not include length of residence in Spain (Model 1) shows that men, young (under 34 years old), Spanish-born, with a high socioeconomic status (monthly incomes of over 1000 euros), who perceive they have adequate social support and low stress, report a better health status.

This research suggests that there is a gradient for age, educational level and income status within the sociodemographic variables considered; individuals reporting poorer health status levels as they get older, also had lower levels of education and income. Additionally, women report poorer self-reported health status than men. This result is consistent with earlier findings in literature on the robust relationship between sociodemographic variables and health status [22], [24], [26], [28], [29]. It also seems other sociodemographic variables such as poor acculturation and discrimination can also affect results [25], [30].

In this study, psychological variables associated with a better self-reported health status were: having good social support (80%) and having no stressful life events (106%).

Additionally, Spanish-born participants reported having better social support and less stress than Latin American-born participants; which is consistent with our earlier findings of Latin American-born and non-Latin American immigrants [31] and, other studies [32].

The single most common reason that Latin American immigrants give for leaving their country of origin is: to seek economic opportunities, improve the future of their family and, provide financial support [33]; this is consistent with the results of this study. Once they have emigrated, a substantial number of them find unsteady work, low pay, oppressive and often physically unsafe economic difficulties, to separation from family and cultural barriers. All of this renders the process of immigration and settlement as very stressful [34], [35]. Our data suggests that the percentage of Latin American-born participants who report feelings of stress is significantly greater than that found for Spanish-born participants. So, stress perceived by the Latin American-born participants as a result of the immigration itself, and of entering and adapting to a new society, is also an important factor to consider along with other variables related to perception of health status. Therefore some sociodemographic and psychosocial variables appears to influence the relationship between immigration status and self-reported health status.

Selection bias is the main limitation in this type of investigation. The study sample was composed of people visiting primary health care centres. This may not be representative of the entire Latin American-born or Spanish-born community. In order to assess the impact of a potential selection bias we compared the numbers of eligible subjects excluded for mental conditions or severe chronic diseases or significant physical or cognitive disabilities were very similar in both populations (figure 1). Furthermore, Fuertes et al. has recently found that for data on visits to primary health care in Spain there is no association between the reason for the visit and nationality, in part due to the very large number of categories in the study sample size [36]. However, the most common reasons for visits to primary health care facilities cited by the Latin America-born and the Spanish-born participants included: respiratory, musculoskeletal and digestive diseases This study did not collect data on the reason for the visit or the real physical comorbidity of the population. Nevertheless, given the low average age of the sample (Mean = 35.9, SD = 10.7), it seems logical to expect a low prevalence of acute or chronic illnesses. The authors believe that if any selection bias exists it is probably small and does not significantly affect the study results. Therefore, we conclude that selection bias is minimal and does not significantly affect the study outcome.

Additionally, this study has a high number of women participants. This is due to the fact that women use primary health care services more often than men [37]. Another limitation is the exclusion of non-Latin American immigrants. The main reason for this was that, according to statistics released by the Spanish Home Office, most immigrants from the north-eastern area of Madrid are Latin Americans; so in general, the sample was representative of the study population. Only including Latin American immigrants ensured all the study participants had a real understanding of the Spanish language. However, not all foreign-born groups are represented in this study; this obliges a cautious interpretation of the data and limits the ability make generalisations about the Latin American-born population. Finally, the cross-sectional design of this study limits the possibility of establishing causal relationships between variables.

Despite the limitations, this research offers an insight into personal and psychosocial factors associated with the self reported health status of Latin America-born and Spanish-born populations. We conclude that a better self-reported health status is associated with: being Spanish-born, men, under 34 years old, with a high-socioeconomic status, having adequate social support, and no stress. The length of time in the host country is a factor to consider in the self-reported health status among immigrant populations. Latin America-born participants who had been living in Spain for less than five years had a better perception of health compared to Latin American participants who have been living in Spain for longer. The self-reported health status of Spanish-born participants is similar to that of Latin American-born participants who have been living in Spain for less than five years, but, is better than that of Latin American-born participants who have been living in Spain for more than five years.
